# Comparison of coronary artery disease guidelines with extracted knowledge from data mining

**DOI:** 10.15171/jcvtr.2017.16

**Published:** 2017-05-22

**Authors:** Peyman Rezaei-Hachesu, Azadeh Oliyaee, Naser Safaie, Reza Ferdousi

**Affiliations:** ^1^Health Information Technology Department, School of Management and Medical Informatics, Road Traffic Injury Research Center, Tabriz University of Medical Sciences, Tabriz, Iran; ^2^Industrial Engineering Faculty, Sharif University Technology, Tehran, Iran; ^3^Cardiovascular Research Center, Tabriz University of Medical Sciences, Tabriz, Iran

**Keywords:** Coronary Artery Disease, Data Mining, Knowledge Discovery, Guideline

## Abstract

***Introduction:*** Coronary artery disease (CAD) is one of the major causes of disability and death in the world. Accordingly utilizing from a national and update guideline in heart-related disease are essential. Finding interesting rules from CAD data and comparison with guidelines was the objectives of this study.

***Methods:*** In this study 1993 valid and completed records related to patients (from 2009 to 2014) who had suffered from CAD were recruited and analyzed. Total of 25 variable including a target variable (CAD) and 24 inputs or predictor variables were used for knowledge discovery. To perform comparison between extracted knowledge and well trusted guidelines, Canadian Cardiovascular Society (CCS) guideline and US National Institute of Health (NIH) guideline were selected. Results of valid datamining rules were compared with guidelines and then were ranked based on their importance.

***Results:*** The most significant factor influencing CAD was chest pain. Elderly males (age >54) have a high probability to be diagnosed with CAD. Diagnostic methods that are listed in guidelines were confirmed and ranked based on analyzing of local CAD patients data. Knowledge discovery revealed that blood test has more diagnostic value among other medical tests that were recommended in guidelines.

***Conclusion:*** Guidelines confirm the achieved results from data mining (DM) techniques and help to rank important risk factors based on national and local information. Evaluation of extracted rules determined new patterns for CAD patients.

## Introduction

Heart disease is the main cause of death in most countries including Iran. Coronary heart diseases and cardiovascular diseases are some classifications of heart diseases.^1^ Of the all global deaths approximately 31% of deaths are related to cardiovascular disease (CVDs) and an estimated 17.5 million people died from CVDs in 2012.^2^ According to Ministry of Health and Medical Education (MoHME)*,* approximately 40% of deaths are due to coronary heart disease, which is considered the most common cause of death in the country. This means that one of every three deaths is due to cardiovascular disease.^3^ Prevention and treatment of heart diseases are becoming a critical concern and global priority.^4,5^ In recent decades, with aid of electronic medical systems, an impressive data amount about cardiovascular factors and diseases have been accumulated in very large databases. There are numerous epidemiological studies have been performed in the literature to identify factors increasing the risk of cardiovascular disease.^6,7^ Today, there are abundant data collected about different diseases with various intentions. The results of this accumulation and integration of data indicate that, the organizations are rich in data, they are weak in deriving knowledge from such data.^8^ This enormous rapid growth of databases in medicine has created great demand for new powerful tools, which turns data into useful, task-oriented knowledge. In efforts to satisfy this need, data mining and knowledge discovery have emerged.^9^ Data mining refers to discovering unknown information and useful patterns from databases.^5^ Knowledge discovery from databases (KDD) consist of several consecutive steps (i.e. problem understanding, data understanding and preparation, data mining (DM), result interpretation and evaluation, and finally using induced knowledge).^7^ The essential step of most knowledge extraction tasks is converting data into knowledge in order to help-decision making, called DM.^10^ There is an increasing tendency for using DM in health related fields such as disease prediction and patient management.^11^ Extraction of knowledge in the form of rules helps physicians in administration of therapeutic process.^12^ DM tasks are focused on identifying rules and relations between features. DM techniques can be used to identify hidden patterns in health related data sets. These patterns can be utilized for clinical diagnosis.^13,14^

Documents that agreed by all members of the medical community called medical guidelines. A clinical guideline, clinical protocol or clinical practice guideline were prepared with the aim of guiding medical decisions. It supports criteria regarding diagnosis, management, and treatment in particular field of healthcare.^15^ With increasing use of information technology in health industry and availability of medical databases over the Internet, accessing to medical guidelines are available for a heart specialist in every step of medical decisions.^16^ Guidelines are great resource of information to make confident medical decisions but international guidelines of pioneer countries require some consideration and changes to be locally beneficial according to ethnic and local situations.^17^ With considering recent advancement in electronic medical records, DM approaches can be applied to local medical data in order to archive localized guidelines. Undeniably, critical importance of using medical guidelines is clear and lead to substantial improvement in quality of medical practice.^18^ In this study we emphasis on capability and importance of DM tasks in localization of medical guidelines. We aimed to investigate the relationships between coronary artery disease (CAD) features, extract the most important risk factors, compare the extracted rules with CAD guidelines, and then prioritize CAD related diagnostic tests based on local information.

## Materials and Methods

### Data acquisition and preparation

The data source was the discharged patients from an academic and educational hospital of Cardiovascular Center in Iran who had admissions for heart disease-related diagnoses during the period from January 01, 2009 to November 30, 2014. From patient groups, only CAD subjects were included in the study. In order to diagnose the presence or absence of CAD, coronary angiography had been performed. Significant CAD was defined as at least one site of 50% or greater diameter stenosis in at least one coronary artery vessel.^19,20^ Minimal CAD or without any stenosis and other cardiovascular diseases were considered as safety groups.

We extracted and constructed a new data set of CAD. Based on literature review, the list of variables associated with CAD was produced and a checklist was developed from the list. The extracted variables were reviewed by a cardiovascular specialist to prepare final features that important for predicting CAD. Table 1 demonstrates 25 features with acceptable class and values that we used in this study to perform DM process. We categorized data values and derived new fields from existing data in the following features: ejection fraction (EF), diastolic blood pressure (DBP), systolic blood pressure (SBP), smoking, triglyceride (Trig), low-density lipoprotein (LDL), high-density lipoprotein (HDL), hemoglobin (Hgb), serum cholesterol, and fasting blood sugar (FBS). These features were changed to categorical attributes for better analysis and getting good results. The data set was highly noisy due to the diversity of patients’ history, physical, and clinical classes. Therefore, we tried efficiently to preprocess the data set using DM preprocessing techniques. Generally, pre-processing of input variables is a vital step in any DM task. We conducted several tasks for creating valid data − elimination of repeated records, fields with spelling errors, fields with additional tokens and other irregularities or irrelevancies. The next step of pre-processing was handling patient records with missing and outlier data. Scaling and coding features are shown in Table 1. After preprocessing, 1993 completed records were extracted and obtained for DM tasks. We partitioned data set into a training set and a testing set; 70% of the data was used for training, and 30% of the data was used for testing.

**Table 1:** Coronary artery disease data set

**Attribute**	**Values**
Pulse rate in beats per minute (BPM)	Numerical (30-150)
Age (y)	Numerical (10-94)
Serum creatinine (mg/dL)	Numerical (0.2-11.6)
Gender	1 = male; 0 = female
Fasting blood sugar (mg/dL)	1 = 70-100; 2 = 101-126; and 3= more than ≥ 127
Serum cholesterol (mg)	1 = ≤ 200; 2 = between 200-239; and 3= above240
Hemoglobin (gm/ml)	1 = ( Hb ≥ 13.5 and Hb ≤ 18 and age >17 and sex = 1) or (Hb ≥ 12 and Hb ≤ 16 and age >17 and sex = 0) or (Hb ≥ 11 and Hb ≤ 16 and age < 17 )= normal; 2 = (Hb < 13.5 and age >17 and sex = 1) or (Hb < 12 and age >17 and sex = 0) or (Hb <= 11 and age < 17 ) = low level; and 3 = ( Hb >18 and age >17 and sex = 1) or ( Hb > 16 and age >17 and sex = 0) or ( Hb > 16 and age < 17 ) = high level
High-density lipoprotein (mg/dL)	1= best ≥60; 2= poor (HDL ≤ 40 and sex = 1) or (HDL ≤ 50 and sex = 0); and 3 = better level ( between 40-59 for men and 50-59 for women)
Low-density lipoprotein (mg/dL)	1= optimal ≤100; 2= near optimal 100-129; 3= border line high 130-159; 4= high 160-189; and 5= very high ≥190
Triglyceride (mg/dL)	1 =less than 150; 2=150-199; 3= 200-499; and 4= ≥ 500
Marital status	0=Single; 1=married
Diabetes mellitus	1= history of diabetes; 0= no such history
Hypertension (mm Hg)	0= no; 1= yes
Family history of coronary disease	0= no; 1= yes
Past history of heart disease	0= no; 1= yes
Dyslipidemia	0= no; 1= yes
Smoker or not	2= current; 3= past; 4= recent; 5= never
Ejection fraction	1= good (50-75); 2=fair(30-49); 3=poor(<30)
Chest pain	2= yes; 3=no
Systolic blood pressure (mm Hg)	1= hypotension =<90; 2=desirable =90-119; 3=border line hypertension =120-139; and 4=hypertension ≥140
Diastolic blood pressure (mm Hg)	1= hypotension <60; 2=desirable = 61-79; 3=border line hypertension =80-89; and 4= hypertension ≥90
Exercise stress test	0= normal; 1= abnormal
Absence or presence of one or more disorders as well as a primary disease	0= no; 1= one comorbidity; 2= more than 2
ST segment and T wave of ECG changes	0= normal; 1= having ST-T wave abnormality
Coronary artery disease diagnosed by physicians	0= no; 1=yes

### Applying data mining techniques

There are various DM techniques available according to their suitability in health care domain. Several DM algorithms that performed successfully in medical fields are used in this research.^21^ Classification is one of the important techniques of DM. Giving a category or class to find antecedently unseen records is the aim of classification.^21^ In order to reach our goals, we used some of the most common predictive DM methods.^22^

**Figure 1:**
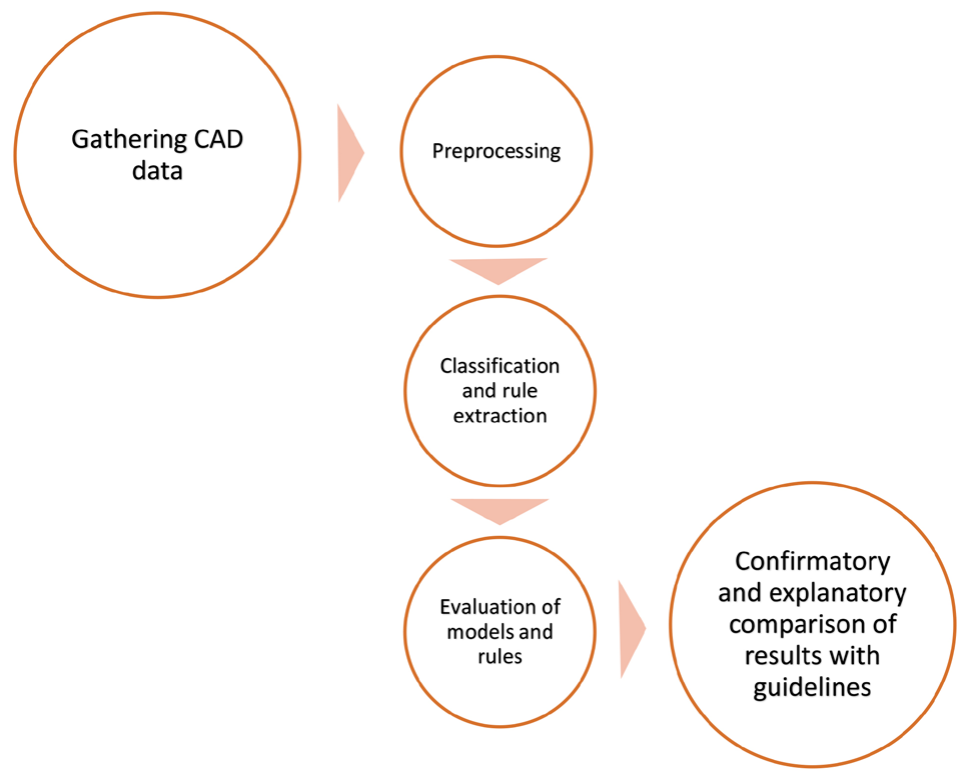
Schematic diagram for the overall steps of the study.

Artificial neural networks (ANNs),^23^ support vector machines (SVM),^24^ Decision tree^25,26^ and ensemble models are algorithms that were used as predictive and analytical methods. ANNs are popular in various areas of medical science.^27-29^ A radial-basis-function (RFB) ANN was employed in this study. SVM has been paid attention in recent years.^22,23^ This algorithm was used to classify CAD data objects with kernel type of RBF and in next phase, decision tree algorithm (C5.0)^30,31^ was developed to classify and extract rules from CAD dataset. Finally, combination of SVM, C5.0 and ANN as an ensemble method was applied. We used SPSS Clementine 12 and CRISP-DM methodology to build mining models. The overall steps of this study is demonstrated in Figure 1.

### Confirmation and comparison of DM results with Guidelines

Canadian Cardiovascular Society (CCS) guideline and US National Institute of Health (NIH) guideline were selected as main references for confirming the results and rules discovered by data mining process. The risk factors and most important signs for CAD extracted from guidelines and then compared with the rules and discovered knowledge from DM process in this study. In the next step diagnostic methods that are provided in guidelines were prioritized according to the obtained results of DM tasks.

## Results

The dataset was composed of 1233 (61.86%) men and 760 (38.13%) women. A total of 1230 (61.71%) ones were patients with CAD and 763 (38.28%) ones were patients without CAD. The relative importance of each variable in estimating the model is associated with the importance of each feature in making a prediction, and it does not relate to model accuracy. The sum of the values for all variables in algorithms is equal to one. SVM, C5.0 and neural network algorithms were used to knowledge discovery. As shown in Table 2, features with great impact on CAD were listed in order of variable importance. Even though each algorithm has assigned different relative weight to features, the most significant factor influencing CAD obtained from all algorithms is chest pain that was included in all induced techniques. Unique variables in each algorithm are seen which are not present in others. For example, the results show that the age variable has only a relative weight of 0.221 in C5.0. Based on these algorithms, other common important risk factors are comorbidities such as lung and digestive disorders, past history of cardiac diseases, FBS, LDL, high blood pressure, sex (especially males), smoking, and low EF. It should be noted that physicians can use this information to analyze the strengths and weaknesses of medical attributes associated with CAD.

**Table 2:** Variable ranking for CAD diagnosis based on data mining approaches

**C C5.0 Important factors** **(relative weight)**	** SVM Important factors** **(relative weight)**	**ANN Important factors** **(Relative weight)**
1-Chest pain (0.421)	1-Chest pain (0.2027)	1-Chest pain (0.1586)
2-Age (0.221)	2-Past history (0.139)	2-Comorbidity (0.1024)
3-Comorbidity (0.220)	3-FBS (0.1255)	3-EF (0.0864)
4-Smoking (0.032)	4-EF (0.1235)	4-Past history (0.0522)
5-Past history (0.02)	5-HgB (0.057)	5-Hgb (0.0481)
6-FBS (0.032)	6-Marital status (0.0551)	6- Diabetes (0.0432)
7-EF (0.027)	7-Cholesterol (0.0498)	7- LDL (0.0413)
8-Triglyceride (0.026)	8-HDL (0.0489)	8-Smoking (0.0413)
9-Hgb (0.023)	9-Diabetes (0.0387)	9-Sex (0.0411)
10-LDL (0.02)	10-Diastolic BP (0.0375)	10-diastolic BP (0.0407)
11-ST (0.018)	11-Smoking (0.0283)	11-ST (0.03782)
12-Sex (0.014)	12-Sex (0.0223)	12-Triglyceride (0.0365)

The extraction of significant rules is presented in this section. Based on the decision tree model (C5.0) with earlier parameter setting, 41 rules were generated with mean estimated accuracy of 84.5%. Only five rules were confirmed by cardiologists (Table 3). However, they emphasized that more investigation with more features and larger data sets is still required. Diagnosis classification rules are interpreted in Table 3 in terms of *if* conditions (antecedent) and conclusion (consequent). The most significant or interesting rules are set in numerical order. Age >54, pulse rate ≤ 88, EF with code 2 and 3 (EF<49), and being a current smoker are considered in CAD diseases.

**Table 3:** Selected rules by cardiovascular specialist

**Rule No.**	**Rules for CAD subjects**
1.	If Chest Pain = yes, past history= yes and comorbidity >1 then CAD=yes
2.	If age > 5‏4 and chest pain = yes and ejection fraction = good (50-75) and ST& T change= having ST-T wave abnormality and sex = male then CAD=yes
3.	If Pulse Rate ≤ 88 and ST& T change= having ST-T wave abnormality then CAD=yes
4.	If age > 5‏4 and chest pain = yes and hemoglobin = low level and sex = male then CAD= 1.0
5.	If age > 69 and past history = yes and smoking = current and pulse rate ≤ 100 then CAD = 1.0

SVM method has the highest accuracy in comparison to the other algorithms. True positive rate (TP / (TP + FN) for SVM, C5.0 and neural network was 0.9541, 0.8239, and 0.6846, respectively. The overall accuracy of SVM was 95.32% in the training set; it was 82.15% in the validation set.

Achieved and evaluated results were compared with risk factors of CAD. According to the CCS, history and all risk factors besides physical examination should be considered. This guideline was prepared for diagnose and management of heart disease. Recommended risk factors mentioned in the CCS are listed in Table 4 and compared with our experimental results. In next step diagnostic tests recommended by US NIH were used to rank the results of DM analyses. Experimental mining results reveals that most important features (except risk factors and historic information) for CAD classifier according to all three algorithms is related to blood test group; FSB, HgB, cholesterol, and HDL.

**Table 4:** Data mining results in comparison with risk factor of CCS guideline about CAD

**Risk factors based on CCS guideline**	**Important Factor base on classifiers**
Tobacco use/smoking history	Confirmed as an important factor
Dyslipidemia	Not confirmed as an important factor for this data set
Diabetes	Confirmed as an important factor
Hypertension	Confirmed as an important factor
Chronic kidney disease	Information not available
lifestyle	Information not available
Age	Confirmed as an important factor
Sex	Confirmed as an important factor
Family history of premature	Confirmed as an important factor
Ethnic origin	Information not available

## Discussion

In this study possibility of using data mining techniques on localization of the medical guidelines in investigated. After acquisition of CAD patient data, we examined different data mining algorithm to identify and ranked the features related to CAD patients, in next step patterns of CAD occurrence extracted in the form of rules. It should be pointed out that all the mining processes reviewed and confirmed by the qualified cardiovascular specialists. Well trusted guidelines confirmed DM findings and finally several diagnostic tests that were recommended in guidelines were prioritized on DM findings. There have been many studies on risk factors of cardiac diseases using DM.^25,32-35^ most of which derived from University of California, Irvine (UCI) data set and may not necessarily apply to local or regional practice. Many risk factors have been associated with CADs. Although different risk factors were obtained from the algorithms investigated, chest pain and past history of cardiac disease were major factors in all methods. We observed that chest pain has the highest effect on CAD presence or absence. Palaniappan and Awang^36^ demonstrated that chest pain is the most significant feature in cardiac patients. While investigating the results, it is worth noting that in all algorithms, patients having chest pain were assumed to be in the CAD subjects group. This represents the highly important effect of chest pain for early diagnosis of cardiac patients. Doctors have also placed great emphasis on chest pain while diagnosing CAD. However, as it was shown, it is noteworthy that absence of chest pain cannot be indicative of healthy coronary arteries.

The results of the study demonstrated that elderly males (age >54) have a high probability to be diagnosed with CAD. This finding is consistent with Tsipouras et al study.^37^ Another important risk factor for CAD in this study was smoking. It has also been validated that smoking has an important role on prediction of CAD in men; 60% of nonsmoker men did not suffer CAD (103/170) and 64% of smokers had CAD (469/730). Jilani et al^5^ showed that the smoking factor contributes significantly to enhance the risk of acute coronary syndrome.

Hypertension, smoking and comorbidity are also extracted by other investigators to have an effect on heart diseases.^33^ Similarly, some features such as age and sex were identified as important in other analyses.^32^

Evaluations of extracted rules determine new patterns for CAD patients. Extracted rules in CAD diseases represented that presence of chest pain, current cigarette smoking, older age, EF rate <50, high blood pressure and comorbidities such as cor pulmonale, pulmonary embolism and hemorrhage have key roles on CAD development. We showed that absence of high blood pressure, moderate level of cholesterol, no smoking and appropriate levels of LDL and HDL were very important factors in maintaining subjects healthy. Therefore, manage stressful situation, have a healthy nutrition, controlling and decreasing blood pressure, appropriately and relatively low levels of LDL, no smoking and tobacco consumption and regular daily activities can help decrease the amount of risk for coronary heart disease. Obviously, in the medical field, diagnoses are basically dependent on physician’s experience; hence some extracted rules may not be accepted easily. However, more effective knowledge and rules are to be obtained by the emergence of new treatment and diagnostic methods.^12^

The CCS guidelines for stable ischemic heart disease (SIHD) updated in 2000^38^ but many advances have since occurred and guidelines from other societies like American college of cardiology updated. Mancini et al^39^ have some complimentary recommendation to update SIHD. So this paper was considered as a base to compare with our results. Most of mentioned risk factors in CCS were highlighted and confirmed in our results. According to National Heart, Lung And Blood Institute (NHLBI) of US NIH, in diagnosing of CAD disease, doctors make decision based on medical history, family history, risk factors and physical exam.^40^ So doctors may need some tests results to diagnose CAD beside risk factors. NIH for this reason recommends six type of tests as follows: electrocardiogram (EKG), stress testing, echocardiography, chest x-ray, blood tests, coronary angiography and cardiac catheterization. Analysis and mining results showed that blood tests have more predictive values among others.

## Conclusion

In this work, valid rules and knowledge were compared with selected guidelines. We have extracted significant rules from the CAD data set for efficient prediction of the disease based on sensitivity and accuracy indicators. Then extracted rules matching with the popular clinical guidelines was investigated. Guidelines in every clinical activity could be important. Mining real and local information about disease and diagnoses helps physicians to weight symptoms based on their experienced importance. They can also choose appropriate medical test to make accurate diagnostic decisions.

Application of DM techniques in analyzing CADs is a good method for investigation of existing relationships between variables. Accurate data, suitable preprocessing and suitable DM technique will offer reasonable results in medical DM.^25^ If risk factors such as hypertension, EF, LDL, cholesterol, smoking and HDL were controlled, CAD risk of a subject may decrease significantly. We believe that these extracted rules could aid as a useful knowledge for physicians in the early prediction of diseases and consequently decrease CAD morbidity. These analyses can be applied to all cardiovascular experts, family physicians, and cardiovascular researchers. Most of the time, clinical decisions are made by physician’s experience, while all physicians are not experienced or expert. Hence, systems with diagnosis support would be a guideline for clinical decision making.^41^ As a result, we believe that general physicians can use this information to perform medical screening just on important attributes instead of doing that on all attributes of patients who are likely to be diagnosed with heart diseases. This will reduce wasting time, medical expenses, administrative costs, and diagnosis time. Moreover, confirmatory and explanatory comparison of extracted rules by data mining techniques with guidelines is helpful in development of more useful and accurate national localized medical guidelines to offer best medical services. It should be pointed out that attributes listed in Table 1 need to be expanded to provide a more comprehensive diagnostics model.

## Ethical Approval

Not applicable.

## Competing interests

The authors declare that there is no conflict of interest.
